# Spine Fragility Fracture Prediction Using TBS and BMD in Postmenopausal Women: A Bayesian Approach

**DOI:** 10.3390/ijerph192114315

**Published:** 2022-11-02

**Authors:** Claudio Ripamonti, Lucia Lisi, Jacopo Ciaffi, Angela Buffa, Renata Caudarella, Francesco Ursini

**Affiliations:** 1 Center for Osteoporosis and Bone Metabolic Diseases, IRCCS Istituto Ortopedico Rizzoli, 40136 Bologna, Italy; 2 Medicine & Rheumatology Unit, IRCCS Istituto Ortopedico Rizzoli, 40136 Bologna, Italy; 3 UOC Medicina Interna ad Indirizzo Reumatologico AUSL, 40133 Bologna, Italy; 4 Azienda USL-IRCCS di Reggio Emilia, 42123 Reggio Emilia, Italy; 5 Casa di Cura Privata Villalba, GVM Care & Research, 40136 Bologna, Italy; 6 Department of Biomedical and Neuromotor Sciences (DIBINEM), Alma Mater Studiorum University of Bologna, 40127 Bologna, Italy

**Keywords:** osteoporosis, TBS, BMD, fracture, fragility, Bayesian

## Abstract

The trabecular bone score (TBS) estimates bone microarchitecture and can be used to evaluate the risk of osteoporotic fractures independently of bone mineral density (BMD). In this retrospective case-control study, we tested and compared the ability of TBS and lumbar spine BMD (LS-BMD) to predict vertebral fragility fractures. The inclusion criteria were female sex, age range 50–90 years, menopause, and clinical risk factors for osteoporosis. Patients with secondary osteoporosis were excluded. LS-BMD and TBS were measured at the L1–L4 vertebral level. The ability of the two diagnostic systems in predicting vertebral fragility fractures was assessed by combining LS-BMD and TBS according to the Bayesian “OR rule” (the diagnosis is negative only for those negative for both tests, and it is positive for those who were positive for at least one test) or to the “AND rule” (the diagnosis is positive only for those positive to both tests and is negative for those negative for at least one test). Of the 992 postmenopausal women included, 86 had a documented vertebral fragility fracture. At the cutoff value used in the present study, the TBS and LS-BMD showed a similar diagnostic ability to predict vertebral fragility fractures, having positive predictive values (PPV) of, respectively, 13.19% and 13.24%. Negative predictive values (NPV) were, respectively, 95.40% and 94.95%. Compared to that of each single diagnostic system, the “OR-rule” significantly increased the NPV to 97.89%, while no statistically significant differences were found by using the “AND-rule”. In conclusion, the present study highlights the possibility that combining LS-BMD and TBS could improve their predictive ability in diagnosing vertebral fragility fractures, and that there is a significant probability of absence of fractures in women who test negative to both diagnostic systems.

## 1. Introduction

The trabecular bone score (TBS) has recently been proposed as a new diagnostic system for assessing the risk of osteoporotic fractures [1]. With TBS, it is possible to estimate bone microarchitecture by using dual-energy X-ray absorptiometry (DXA) [2,3]. It has also been reported that the TBS predicts fracture risk independently of bone mineral density (BMD) [4,5,6]. The combined use of TBS and BMD has also been shown to be superior than each diagnostic system alone to obtain a reliable estimate of the risk of fracture [7,8], albeit with some exceptions [9]. In other studies, the TBS has been shown to differentiate fractures even in osteopenic subjects [5,9,10,11,12,13,14], proposing itself as a useful diagnostic tool for redefining fracture risk among women who are not classified as osteoporotic by BMD, which is currently the gold-standard method for diagnosing osteoporosis [15]. A majority of papers evaluating the ability of TBS to predict fragility fractures have used frequentist statistics with odds ratio to assess the overall fracture discriminant ability of the test. Bayesian statistics, which also individually evaluates the probability of positive and negative diagnostic results associated or not with fracture [16], has rarely been used. In this study, we used a Bayesian statistical approach to investigate the ability of TBS to estimate the probability of spine fragility fractures in the overall sample of postmenopausal women included, and also in those who tested negative to the spine BMD. Finally, we evaluated whether the combination of TBS with lumbar spine BMD (LS-BMD) improved the ability of LS-BMD alone to estimate the probability of spine fragility fractures.

## 2. Materials and Methods

The data used in this retrospective case-control study were an extension of the casuistry the authors had used in a previous publication [14]. Briefly, the study enrolled women who were in- or out-patients of the Rizzoli Orthopaedic Institute, Bologna, Italy, who were referred for DXA owing to clinical risk factors for osteoporosis. Selection criteria included menopause, the presence of vertebral fragility fractures due to minor trauma, and/or the presence of clinical risk factors for osteoporosis, as well as an age range of 50–90 years. Exclusion criteria were the presence of diseases or the chronic intake of drugs known to cause secondary osteoporosis; severe obesity or thinness (BMI > 35 Kg/m^2^ or <17 Kg/m^2^); and previous fractures other than spine fragility fracture. Only women with spine fragility fractures documented by radiologic vertebral crushing were included in the study, together with women having clinical risk factors for osteoporosis but not reporting fractures. The required information was gathered from the applications filed by the physicians applying for the DXA. The results of LS-BMD (g/cm^2^) and TBS analyses, age, age at menopause, weight and height were recorded at the time of DXA. DXA of the L1–L4 vertebrae was carried out by using the technique described by the densitometer manufacturer. Only DXA acquisitions, which were performed by Discovery QDR (Hologic.INC, Bedford, MA, USA), without accuracy errors according to the judgement of the examiner, were used in the study. Trabecular bone score iNsight (Med-Imaps TBS version 1.9.1) software was used to calculate the TBS score from the L1–L4 DXA. Statistical analyses were performed by using Bayesian statistics, focusing on the role of TBS alone or combined with the LS-BMD in the assessment of osteoporotic fragility fractures in clinical practice [17]. The research was conducted in compliance with the Declaration of Helsinki and its latest amendments [18]. The study was approved by the local Ethics Committee (Comitato Etico Area Vasta Emilia Centrale, Bologna, Italy—approval number: 0005003, date 2 April 2020).

### Statistical Analysis

The data analysis was carried out by using SPSS version 11 software (SPSS/PC, Chicago, IL, USA). For the statistical elaboration, data were reported as mean and standard deviation (SD) for continuous variables. The unpaired Student’s *t*-test was used for comparison between groups of variables selected after verifying normal distribution and homogeneity of variance (Levene test). The association of factures with diagnostic tests was obtained by using 2 × 2 contingency tables (CTs), which were subsequently used, when appropriate, to calculate the diagnostic accuracy parameters of the TBS and LS-BMD, according to the methods of Bayesian statistics. The overall accuracy of the diagnostic systems was measured by using odds ratio (OR) and 95% confidence interval (95% CI) [19]. This was calculated by using the Mantel–Haenszel test as the ratio of the fractures testing positive (positive odds) divided by the ratio of the fractures testing negative (negative odds). The *Z*-test was used for the OR comparison after Log transformation of the proportion. The covariates of the receiver operating characteristic (ROC) curves, having fracture as the state variable, and LS-BMD or TBS as the test variable, were used to fix the diagnostic cutoffs of each test in the study, utilizing the maximum of the Youden index. The values of the TBS or LS-BMD greater than the respective cutoffs were defined to be negative tests, and those lower than or equal to the cutoff were defined to be positive tests. The ability of the diagnostic systems to correctly classify women with fractures (discriminative power) was measured by using sensitivity (SE) and specificity (SP), together with their confidence intervals calculated by “exact” Clopper–Pearson confidence intervals [20]. The between-tests comparison of the SE and SP were carried out by comparing the likelihood ratio in pair design [21]. The ability of the diagnostic systems to predict the post-test probability of fracture (predictive ability) was assessed by calculating the positive predictive value (PPV) (% of women having spine fractures over the total number of women positive to the diagnostic test) and the negative predictive value (NPV) (i.e., the % of women not having spine fractures over the total number of women negative to the diagnostic test). Fracture prevalence (or pre-test probability) was calculated by dividing the number of women with fractures by the total number of women in the study sample. The CIs of the PPV and the NPV were calculated according to Mercaldo et al. [22]. The Kosinski test was adopted to compare the *PPVs* and the *NPVs* of the two diagnostic tests [23]. The SE, SP, PPV, and NPV resulting from the combination of the two diagnostic tests were calculated according to the “OR rule” and the “AND-rule”, which were obtained by using the dedicated equations of Bayesian statistics. The “OR-rule” considers the diagnosis to be negative only for those negative for both tests, and to be positive for those who were positive for at least one diagnostic test. The “AND-rule” considers only those positive for both tests to be positive and those negative for at least one test to be negative [24]. Cohen’s kappa statistics were used to assess the degree of concordance between the dichotomous qualitative variables of positivity or negativity to LS-BMD or to TBS in the study population. The correlations between the percentages of the pre-test fracture prevalence (at different pre-test fracture probability) and the PPVs of the “AND-rule” and the NPVs of the “OR-rule” were tested by linear regression.

## 3. Results

In our study, we included 992 women who met the criteria from a sample of 1513 postmenopausal women; therefore, 521 patients were excluded. The mean age of the included patients was 68.5 ± 6.8 years (range 51–90 years). Fracture prevalence was 8.67%. The comparison between those with fractures and those without fractures is reported in Table 1.

The women with fractures were significantly shorter, younger at menopause, and had lower TBS and LS-BMD values. The diagnostic fracture threshold, calculated on the entire sample of women selected, corresponded to an LS-BMD value of 0.800 g/cm^2^ (T-score of −2.3) and to a TBS of 1.204. All the statistical analyses in the study were carried out at those cutoffs, when appropriate.

### 3.1. Diagnostic Concordance between the Two Tests

In the entire study sample, the women’s diagnostic classifications, which were carried out by using the TBS and LS-BMD, had poor concordance at the K Cohen Test (K 0.355; Effect Size (ES) 0.030) (Table 2).

When analyzing women with fractures and women without fractures separately, agreement between the two diagnostic tests in classifying women positive or negative was poor in the women with fractures (Cohen’s K = 0.100) and in those without fractures (Cohen’s K = 0.367) (Table 3).

### 3.2. Diagnostic Accuracy Measurement of the TBS and LS-BMD in the Entire Sample of Women (Fracture Prevalence 8.67%)

The TBS ranked 52.6% of the women as negative and 47.4% as positive. The statistical analysis showed a PPV of 13.19% (95% CI: 11.48–14.91), an NPV of 95.40% (95% CI: 93.63–96.70), an SE of 72.09% (95% CI: 61.38–81.23), and an SP of 54.97% (95% CI: 51.66–58.24). The fracture OR was 3.15 (95% CI 1.93–5.14) (Chi square *p* < 0.001) (Table 4). When the same women were tested by using LS-BMD, 44.20% tested positive and 55.80% tested negative. Statistical analysis showed a PPV of 13.24% (95% CI: 11.34–15.15), an NPV of 94.95% (95% CI: 93.46–96.43), an SE of 67.44% (95% CI: 56.48–77.16), and an SP of 58.06% (95% CI: 54.77–61.30). The fracture OR was 2.87 (95% CI: 1.79–4.59). The Chi-Square test for OR significance was *p* < 0.001 (Table 4). There were no statistically significant differences between the percentages of the diagnostic accuracy parameters considered between the TBS and the LS-BMD (Table 4).

### 3.3. Diagnostic Accuracy Measurement of TBS in the Women Who Tested Negative to LS-BMD (Fracture Prevalence of 5.05%)

Of the women who were LS-BMD negative, the TBS classified 31.59% as testing positive and 68.41% as testing negative. In this subgroup of women, the SE was 71.43% (95% CI: 51.12–86.05) and the SP was 70.53% (95% CI: 66.40–74.36). The PPV was 11.19% (95% CI: 8.01–14.37) and the NPV was 97.89% (95% CI: 96.72–99.06). The OR was 5.98 (95% CI: 2.58–13.88), which was statistically significant with *p* < 0.0001 at the Chi-Square test.

### 3.4. Diagnostic Accuracy Measurement of LS-BMD in the Women Who Tested Negative to the TBS (Fracture Prevalence 4.60%)

LS-BMD classified the TBS negative women as positive in 27.39% of cases and as negative in 72.61% of cases. In the subgroup of TBS negative women, the LS-BMD had a SE of 66.67% (95% CI 43.11–84.52) and a SP of 74.50% (95% CI: 70.21–78.47), together with a PPV of 1.43% (95% CI: 8.71–14.15%) and a NPV of 97.89% (95% CI: 94.67–99.10). The fracture OR was 5.84 (95% CI: 2.44–13.98), which was statistically significant (Chi Square: *p* < 0.0001).

### 3.5. Diagnostic Accuracy Measurement of the Entire Sample of Women (Fracture Prevalence 8.67%), Combining the Two Tests According to the “OR-Rule” (Fracture Prevalence 8.67%)

When combining the TBS and LS-BMD according to the “OR rule”, 61.79% of the diagnostic tests were positive, and 38.21% were negative. The SP of the “OR rule” 41.00% (95% CI: 37.90–44.29) was significantly lower than that of each of the two tests considered individually (Wald test: *p* = 0.004 for the TBS and *p* < 0.001 for the LS-BMD). The SE 90.70%; (95% CI: 78.60–91.10) of the “OR rule” was significantly greater than that of the TBS (Wald test: *p* < 0.001) and the LS-BMD (Wald test: *p* < 0.001). There was no statistically significant difference between the PPV of the “OR-rule” 12.73% (95% CI: 11.76–13.69), and those of the TBS and the LS-BMD (Wald test: *p* = 0.750 and *p* = 0.524, respectively). The NPV of the “OR rule” 97.89% (95% CI: 96.52–99.26) was significantly greater than that of the LS-BMD (Wald test: *p* = 0.002) and that of the TBS (Wald test: *p* < 0.001). The false positives and false negatives were 87.27% (95% CI: 84.64–89.91) and 2.11% (95% CI; 0.66, 3.56), respectively. The fracture OR 6.76 (95% CI: 3.23–14.17) of the combination according to the “OR rule” was statistically significant (Pearson chi square test: *p* < 0.001). It was superior—but not statistically significantly so—to that of the LS-BMD (*Z*-test on Log transformed results, *p* = 0.063) and the TBS (*Z*-test on log transformed results, *p* = 0.096).

### 3.6. Diagnostic Accuracy Measurement of the Entire Sample of Women, Combining the Two Tests According to the “AND-Rule” (Fracture Prevalence 8.67%)

When carrying out the analysis by using the “AND-rule”, 29.73% of the diagnostic tests were positive, and 70.27% were negative. The SE 48.84% (95% CI: 38.67–59.34) of the “AND-rule” was lower than those of the TBS (Wald test: *p* < 0.001) and the LS-BMD (Wald test: *p* < 0.001) individually. The SP of the “AND-rule” was 72.08%; (95% CI: 68.81–74.64) was greater than those of each of the two tests considered individually (in both cases, Wald test: *p* < 0.001). The PPV of the “AND-rule” was 14.24% (95% CI: 11.30–17.17) and the NPV was 93.69% (95% CI: 92.44–94.93). The PPV was not significantly different from those of the LS-BMD and the TBS (Wald test: *p*= 0.622 and *p* = 0.647, respectively). The NPV of the “AND rule” was lower, but not significantly so, than those of the TBS and the LS-BMD (Wald test: *p* = 0.091 and *p* = 0.177, respectively). The fracture OR 2.46; (95% CI: 1.58–3.85) of the “AND rule” combination was statistically significant (Pearson chi square test: *p* < 0.001); however, it was not significantly different from that of each of the two tests considered individually (*Z*-test on Log transformed results; LS-BMD: *p* = 0.305, TBS: *p* = 0.359).

### 3.7. Calculation of the Post-Test Probability of Fracture at Different Percentages of the Pre-Test Probability

**Figure 1:**
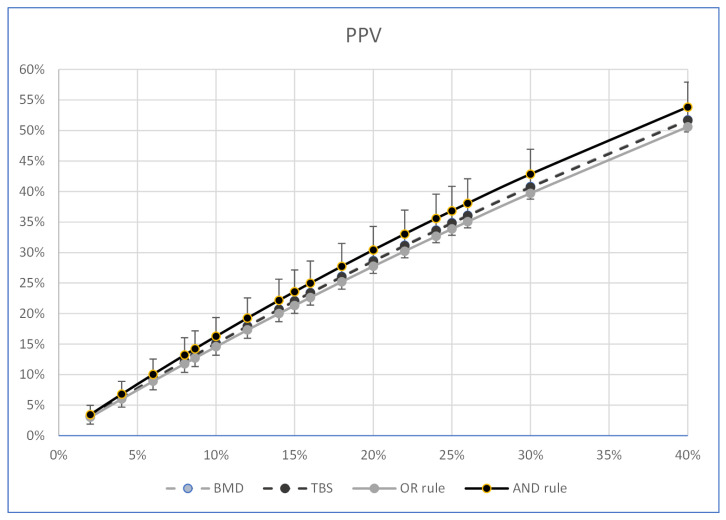
Graph showing the trend of the PPVs (ordinates) of the LS-BMD, the TBS, the “OR rule”, and the “AND rule” as the fracture prevalence values varied (abscissas).

**Figure 2:**
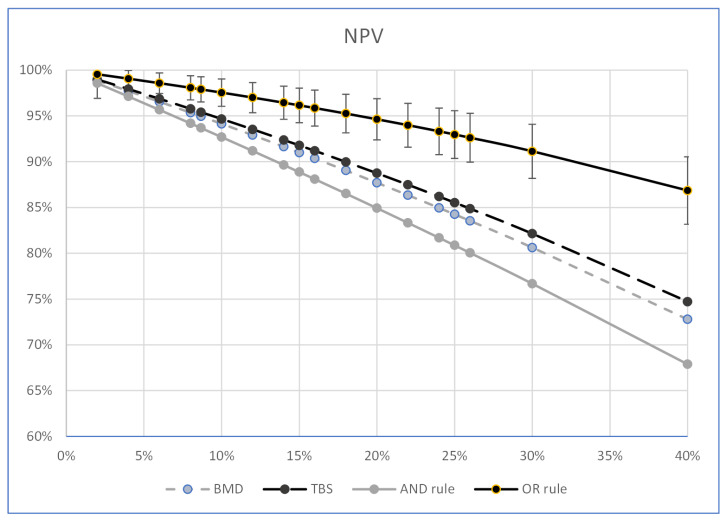
Graph showing the NPV trend of the LS-BMD, the TBS, the “OR rule”, and the “AND rule” (ordinates) as the fracture prevalence values varied (abscissas).

Table 5 and Table 6 show and compare the PPV and NPV percentages of the “OR-rule”, the “AND-rule”, the LS-BMD, and the TBS. These percentages were calculated at the pre-test probability of the sample selected for the study (8.67%) and at other pre-test probability percentages from 2 to 40%. All the calculations of the post-test probability were carried out at the values of SE and SP considered in the study, using the number of women in the present sample. The two diagnostic systems combined according to the “AND-rule” had better PPV percentages than those of each diagnostic system considered individually; however, the difference was not statistically significant (Table 5 and Figure 1). The combination of the two diagnostic systems according to the “OR rule” (Table 6, Figure 2) showed the best percentages of the NPVs at each pre-test probability value considered; they were greater than those of the “AND rule” (*p* = 0.001, using the Kosinski method with the Bonferroni correction) at all the estimated probability percentages, and greater than those of the individual LS-BMDs and TBSs with a difference which, above the pre-test prevalence of 6%, became statistically significant (at a fracture prevalence of 6%, the statistical significance, calculated by using the Kosinski method with the Bonferroni correction, was *p* = 0.023 for the LS-BMD and *p* = 0.054 for the TBS). The R and R2 of the correlation between the percentages of the pre-test fracture prevalence and the PPVs of the “AND rule” were 0.997 (*p* < 0.001), and 0.994 (*p* = 0.001), respectively, and those of the correlation between the percentages of the pre-test fracture prevalence and the NPVs of the “OR rule” were −0.993 (*p* < 0.001) and −0.987 (*p* = 0.001), respectively.

## 4. Discussion

Using Bayesian statistics, the ability to predict vertebral fragility fractures of two diagnostic systems (i.e., TBS and LS-BMD) was tested in a group of 992 post-menopausal women. Attention was focused on three points: the comparative ability of the TBS and LS-BMD used individually to predict vertebral fragility fractures, the ability of each of them to predict the presence of vertebral fractures only in women who tested negative when using other diagnostic systems, and, finally, the ability of LS-BMD and the TBS combined to predict spine fracture.

At the cutoff value used in the present study, the TBS and LS-BMD showed similar overall diagnostic ability to predict women with vertebral fracture in accordance with some studies [5,9,10]; however, it was in contrast to others [7,11,13]. The estimated SEs, SPs, PPVs, and NPVs were not significantly different between the LS-BMD and the TBS. In particular, it was found that the PPV was lower than that of the NPV in both diagnostic systems, documenting a better ability to predict true negatives than true positives. This was analogous to other reports in the literature which used Bayesian statistics [4,20,21,22,23,25]. The relatively low pre-test prevalence of vertebral fragility fractures of the majority of this type of study could, at least in part, justify this finding. In fact, the PPV and the NPV were directly and indirectly related to the pre-test probability of disease, respectively [26]; this influenced their respective predictive abilities.

After using the K Cohen test to verify that the diagnostic concordance between the TBS and the LS-BMD was poor, and that, therefore, the LS-BMD and the TBS predicted a part of fractures differently, the ability of the TBS to predict fractures among women testing negative to LS-BMD was evaluated.

The TBS classified 31.59% of the women who tested negative to LS-BMD as positive; of these, 23.3% sustained fractures. These data confirmed those of other authors regarding the overall ability of the TBS to predict fractures, even among women having an LS-BMD outside the range of osteoporosis [7,9,10,11,27]. In addition to the PPVs and NPVs of the TBS, it was found that, by analyzing the women who tested negative to the LS-BMD, other data in the literature were confirmed. Moreover, calculated at the same fracture prevalence, they were similar to those reported by Albrecht W. et al. [4] regarding osteopenic women. Similar results were also obtained by testing the TBS-negative women with LS-BMD, confirming the diversity of bone structural factors which could be detected by using each single method.

The fact that the TBS classified some women with fractures who were LS-BMD negative as positive led to the belief that, when combining LS-BMD and the TBS, a larger number of women with fractures would be detected as positive. By so doing, women could be considered to be positive when they were positive to only one or the other of the two diagnostic systems. This effectively occurred when the Bayesian “OR rule” was applied. In fact, when using the “OR rule” in the present sample, it was observed that the SE value became greater than those of each individual diagnostic system, indicating that combining LS-BMD and the TBS allowed classifying a larger percentage of women with fractures as positive. However, it should be noted that the improved sensitivity for fractures resulting from the combined use of LS-BMD and the TBS was not necessarily associated with an improvement in their ability to differentiate those with fractures from those without [28]. In fact, in the present study, the PPV of the “OR rule” was even lower than that of the LS-BMD since the false positive rate increased parallelly. The total diagnostic performance of the “OR-rule”, however, was better than that of each individual diagnostic system in agreement with other studies [5,7], and its OR ratio even approached statistical significance regarding the LS-BMD. The high percentage of the NPV value, which was statistically significant versus both the LS-BMD and the TBS certainly contributed to the better total diagnostic performance of the two systems combined according the “OR rule”. When using the “OR rule”, the NPV became reliable enough to consider the presence of spine fragility fractures in women who were negative to both diagnostic systems highly improbable. Unfortunately, the “AND-rule” did not significantly improve the overall diagnostic ability of the two diagnostic systems considered individually, nor did it improve their positive and negative post-test fracture probability. In addition, it should be noted that the SE of the “AND rule” was also lower than that of each individual test, leading to think that the combined positivity to the LS-BMD and the TBS was not useful in improving the ability of each diagnostic system to detect fractures.

The PPV and NPV of the TBS, the LS-BMD and of their combination at different fracture pre-test probabilities were then calculated to look at the resulting variations of those parameters. The “OR rule” showed that the NPV of the women who are negative to both diagnostic systems was higher than those estimated by the LS-BMD or the TBS individually at all the fracture prevalence values tested, with a gap above the pretest probability of 6%, which became statistically significant (Figure 2). Notably the predictive ability of the NPV of the “OR rule” had statistically significant values for fracture prevalence greater than those generally reported in free-living postmenopausal women [29]. On the contrary, the “AND rule” showed that women positive to both diagnostic systems had low PPV values at all the fracture prevalences in the present study; this did not significantly change those estimated by each individual diagnostic system.

To the authors’ knowledge, the only study in the literature which allowed a comparison with the present data regarding the percentage of PPVs and NPVs when the LS-BMD and the TBS were combined according to Bayesian statistics was that of Nassar et al. [10]. The present results confirmed those of these authors. In fact, the present data, recalculated at the pre-test probability of the Nassar study for homogeneity of comparison, showed similar percentages of the NPV at the “OR-rule” in women testing negative and of the PPV at the “AND-rule” for those testing positive.

In summary, according to the present data, LS-BMD and the TBS predicted fracture to a similar extent; however, the identification of true-positive women was partially discordant between the two diagnostic systems. Compared to that of each diagnostic system, according to the “OR rule”, their combination allowed us to identify a greater number of women with vertebral fractures (increase SE); however, at the same time, there was a greater number of false positives, leading to a low rate of correct positive-fracture prediction (PPV did not increase). Using the “AND rule”, the women positive to both diagnostic systems did not have a better sensitivity or positive predictive value for fracture than those who tested positive at each individual diagnostic system; finally, the probability of not being fractured in women testing negative to both diagnostic systems was greater than those estimated by LS-BMD and the TBS individually.

Since the simultaneous negativity to both diagnostic systems gave a strong probability of fracture absence, it appeared that, when searching for women negative to both diagnostic systems, the TBS can simply be used as a second investigation in subjects negative to LS-BMD in order to confirm their negativity and their low probability of fracture [5].

The present study had several limitations. In fact, it was limited to vertebral fractures diagnosed following investigations required for clinical symptoms already in place. It was not accompanied by other analyses aimed at ascertaining the metabolism of the bone, and it was carried out on DXA women who were referred due to having risk factors for osteoporosis.

## 5. Conclusions

In conclusion, the present study highlights the possibility that combining LS-BMD and TBS could improve the overall predictive ability of DXA in diagnosing vertebral fragility fractures and that there is a significant probability of absence of fractures in women who test negative to both diagnostic systems. Additional research is, nevertheless, necessary to confirm the present results.

## Figures and Tables

**Table 1:** Baseline characteristics of the 992 women included in the study and a comparison between women with fractures and women without fractures.

	Whole Population (*n =* 992)	Women with Fractures (*n =* 86)	Women without Fractures (*n =* 906)	*p*-Value
Age (years), mean ± SD	68.5 ± 6.8	69.6 ± 6.8	68.4 ± 6.8	0.140
Height (cm), mean ± SD	159.5 ± 6.2	158.1 ± 6.4	159.6 ± 6.1	0.032
Weight (kg), mean ± SD	63.3 ± 10.4	62.6 ± 10.6	63.4 ± 10.4	0.501
BMI, mean ± SD	24.9 ± 3.7	25.0 ± 4.1	24.8 ± 3.7	0.553
Age at menopause (years), mean ± SD	49.4 ± 4.7	48.4 ± 5.5	49.5 ± 4.6	0.046
TBS, mean ± SD	1.210 ± 0.101	1.165 ± 0.095	1.214 ± 0.100	0.001
LS-BMD (g/cm^−2^), mean ± SD	0.823 ± 0.122	0.771 ± 0.127	0.828 ± 0.120	0.001
LS-BMD T-score, mean ± SD	−2.02 ± 1.11	−2.49 ± 1.15	−1.98 ± 1.09	0.001

Legend: BMI, body mass index; LS-BMD, lumbar spine bone mineral density; SD, standard deviation.

**Table 2:** Diagnostic concordance between the TBS and LS-BMD.

	TBS Positive %	TBS Negative %	Total %
LS-BMD Positive	29.8	14.4	44.2
LS-BMD Negative	17.6	38.2	55.8
Total	47.4	52.6	100

**Table 3:** Diagnostic concordance between the TBS and LS-BMD in classifying women positive or negative by analyzing women with and without fractures separately (K Cohen test).

	Women with Fractures			Women without Fracturs		
	TBS Positive %	TBS Negative %	Total %	TBS Positive %	TBS Negative %	Total %
LS-BMD Positive test	48.8	18.6	67.4	27.9	14.0	41.9
LS-BMD negative test	23.3	9.3	32.6	17.1	41.0	58.1
Total	72.1	27.9	100	45.0	55.0	100

K Cohen for the women with fractures = 0.010 (poor concordance). K Cohen for the women without fractures = 0.367 (poor concordance).

**Table 4:** Diagnostic accuracy measurements of the TBS, and of LS-BMD calculated in the entire sample of the 992 women in the study (cutoff values = 1.204 for the TBS, and 0.800 g/cm^2^ for LS-BMD) and their comparison. The pre-test fracture prevalence was 8.67% (95% CI: 6.99–10.60).

	TBS Diagnostic Accuracy Values	LS-BMD Diagnostic Accuracy Values	*p*-Value TBS vs. LS-BMD
SE% (95% CI)	72.09 (61.38–81.23)	67.44 (56.48–77.16)	*ns* ($\chi_{WSe}^{2}$ = 0.446)
SP% (95% CI)	54.97 (51.66–58.24)	58.06 (54.77–61.30)	0.09 ($\chi_{WSp}^{2}$ = 2.788)
PPV% (95% CI)	13.19 (11.48–14.91)	13.24 (11.34–15.15)	ns (*TV_pp_ WGS* = 0.001)
NPV% (95% CI)	95.40 (93.63–96.70)	94.95 (93.24–96.24)	ns (*TV_pn_ WGS* = 0.16)
OR (95% CI)	3.15 (1.93–5.14)	2.87 (1.79–4.59)	ns

Legend: LS-BMD, lumbar spine bone mineral density; NPV, negative predictive value; ns, non-significant; OR, odds ratio; PPV, positive predictive value; SE, sensitivity; SP, specificity; TBS, trabecular bone score. Confidence intervals for SE, SP, and accuracy are “exact” Clopper–Pearson confidence intervals. Confidence intervals for the PPVs and the NPVs are Wald-type intervals.

**Table 5:** PPV average percentage value (and relative CIs) of the LS-BMD, the TBS, the “OR rule”, and the “AND rule”, calculated at different fracture prevalence values and at the same SE and SP values used in the study.

	BMD			TBS			OR Rule			AND Rule		
Prevalence	PPV (95% CI)			PPV (95% CI)			PPV (95% CI)			PPV (95% CI)		
2.0%	3.2%	3.0%	6.5%	3.2%	3.0%	6.3%	3.0%	2.7%	5.6%	3.4%	1.9%	5.0%
4.0%	6.3%	5.4%	10.0%	6.3%	5.4%	9.8%	6.0%	5.4%	6.7%	6.8%	4.7%	8.9%
6.0%	9.3%	7.7%	10.9%	9.3%	7.8%	10.7%	8.9%	8.1%	9.7%	10.0%	7.5%	12.6%
8.0%	12.3%	10.4%	14.1%	12.2%	10.6%	13.9%	11.8%	10.9%	12.7%	13.2%	10.4%	16.0%
8.67%	13.2%	11.3%	15.1%	13.2%	11.5%	14.9%	12.7%	11.8%	13.7%	14.2%	11.3%	17.2%
10.0%	15.2%	13.1%	17.2%	15.1%	13.3%	16.9%	14.6%	13.5%	15.6%	16.3%	13.2%	19.4%
12.0%	18.0%	15.8%	20.2%	17.9%	16.0%	19.9%	17.3%	16.2%	18.5%	19.3%	16.0%	22.6%
14.0%	20.7%	18.4%	23.0%	20.7%	18.6%	22.8%	20.0%	18.8%	21.2%	22.2%	18.7%	25.6%
15.0%	22.1%	19.7%	24.5%	22.0%	19.9%	24.2%	21.3%	20.0%	22.6%	23.6%	20.0%	27.1%
16.0%	23.4%	21.0%	25.9%	23.4%	21.2%	25.6%	22.6%	21.3%	24.0%	25.0%	21.4%	28.6%
18.0%	26.1%	23.6%	28.6%	26.0%	23.7%	28.3%	25.2%	23.8%	26.6%	27.7%	24.0%	31.5%
20.0%	28.7%	26.1%	31.3%	28.6%	26.2%	30.9%	27.7%	26.3%	29.2%	30.4%	26.6%	34.3%
22.0%	31.2%	28.5%	33.9%	31.1%	28.7%	33.5%	30.2%	28.7%	31.8%	33.0%	29.1%	36.9%
24.0%	33.7%	31.0%	36.4%	33.6%	31.1%	36.1%	32.7%	31.1%	34.3%	35.6%	31.6%	39.6%
25.0%	34.9%	32.1%	37.6%	34.8%	32.3%	37.3%	33.9%	32.3%	35.5%	36.8%	32.8%	40.8%
26.0%	36.1%	33.3%	38.9%	36.0%	33.5%	38.5%	35.1%	33.4%	36.7%	38.1%	34.0%	42.1%
30.0%	40.8%	38.0%	43.6%	40.7%	38.1%	43.3%	39.7%	38.0%	41.4%	42.8%	38.8%	46.9%
40.0%	51.7%	48.8%	54.6%	51.6%	48.9%	54.3%	50.6%	48.7%	52.4%	53.8%	49.8%	57.9%

The comparisons of the PPVs were carried out vs. the “AND-rule”, using the Kosinski method with the Bonferroni correction; there was no statistically significant comparison with the PPVs of the “AND rule”, the LS-BMD, and the TBS.

**Table 6:** NPV average percentage values (and relative CIs) of the LS-BMD, the TBS, the “OR rule”, and the “AND rule” calculated at different fracture prevalence values and at the same SE and SP values used in the study.

	BMD			TBS			OR Rule			AND Rule		
Prevalence	NPV (95% CI)			NPV (95% CI)			NPV (95% CI)			NPV (95% CI)		
2.0%	98.9%	96.7%	98.6%	99.0%	96.8%	98.6%	99.5%	97.0%	98.6%	98.6%	98.0%	99.2%
4.0%	97.7%	95.3%	97.8%	97.9%	95.4%	97.9%	99.1%	98.2%	100.0%	97.1%	96.3%	98.0%
6.0%	96.5%	95.3%	97.8%	96.9%	95.6%	98.1%	98.6%	97.4%	99.7%	95.7%	94.6%	96.7%
8.0%	95.4%	93.9%	96.8%	95.8%	94.3%	97.2%	98.1%	96.7%	99.4%	94.2%	93.0%	95.4%
8.67%	94.9%	93.5%	96.4%	95.4%	93.9%	96.9%	97.9%	96.5%	99.3%	93.7%	92.4%	94.9%
10.0%	94.1%	92.5%	95.7%	94.7%	93.0%	96.3%	97.5%	96.1%	99.0%	92.7%	91.4%	94.0%
12.0%	92.9%	91.2%	94.6%	93.5%	91.7%	95.3%	97.0%	95.3%	98.6%	91.2%	89.7%	92.6%
14.0%	91.6%	89.7%	93.5%	92.4%	90.4%	94.3%	96.4%	94.6%	98.2%	89.6%	88.1%	91.2%
15.0%	91.0%	89.0%	92.9%	91.8%	89.8%	93.8%	96.1%	94.3%	98.0%	88.9%	87.3%	90.5%
16.0%	90.3%	88.3%	92.4%	91.2%	89.1%	93.3%	95.9%	93.9%	97.8%	88.1%	86.4%	89.7%
18.0%	89.0%	86.9%	91.2%	90.0%	87.8%	92.2%	95.3%	93.1%	97.4%	86.5%	84.8%	88.3%
20.0%	87.7%	85.5%	90.0%	88.7%	86.4%	91.1%	94.6%	92.4%	96.9%	84.9%	83.1%	86.8%
22.0%	86.3%	84.0%	88.7%	87.5%	85.0%	89.9%	94.0%	91.6%	96.4%	83.3%	81.4%	85.2%
24.0%	85.0%	82.5%	87.4%	86.2%	83.6%	88.7%	93.3%	90.8%	95.8%	81.7%	79.7%	83.7%
25.0%	84.3%	81.7%	86.8%	85.5%	82.9%	88.1%	93.0%	90.4%	95.6%	80.9%	78.9%	82.9%
26.0%	83.5%	81.0%	86.1%	84.9%	82.2%	87.5%	92.6%	89.9%	95.3%	80.0%	78.0%	82.1%
30.0%	80.6%	77.9%	83.4%	82.1%	79.3%	85.0%	91.1%	88.2%	94.1%	76.7%	74.5%	78.8%
40.0%	72.8%	69.7%	75.9%	74.7%	71.4%	78.0%	86.8%	83.2%	90.5%	67.9%	65.5%	70.2%

The NPV comparisons were carried out vs. the “OR rule” using the Kosinski method with the Bonferroni correction: *p* value: *p* < 0.01 with the “AND rule” for all prevalences; *p* = 0.023 with the LS-BMD, and *p* = 0.054 with the TBS at a prevalence of 6% for both diagnostic tests.

## Data Availability

The data that support the findings of this study are available from the corresponding author, J.C., upon reasonable request.
